# *Enterococcus faecalis* Endocarditis and Outpatient Treatment: A Systematic Review of Current Alternatives

**DOI:** 10.3390/antibiotics9100657

**Published:** 2020-09-30

**Authors:** Laura Herrera-Hidalgo, Arístides de Alarcón, Luis E. López-Cortes, Rafael Luque-Márquez, Luis F. López-Cortes, Alicia Gutiérrez-Valencia, María V. Gil-Navarro

**Affiliations:** 1 Unidad de Gestión Clinica de Farmacia, Hospital Universitario Virgen del Rocío/CSIC/Instituto de Biomedicina de Sevilla (IBiS), 41009 Seville, Spain; laura.herrera.sppa@juntadeandalucia.es (L.H.-H.); mariav.gil.sspa@juntadeandalucia.es (M.V.G.-N.); 2 Unidad de Gestión Clinica de Enfermedades Infecciosas, Microbiología y Medicina Preventiva, Hospital Universitario Virgen del Rocío/CSIC/Instituto de Biomedicina de Sevilla (IBiS), 41009 Seville, Spain; caristo.alarcon.sspa@juntadeanalucia.es (A.d.A.); rafael.luque.sspa@juntadeandalucia.es (R.L.-M.); lflopez@us.es (L.F.L.-C.); 3 Unidad de Gestión Clinica de Enfermedades Infecciosas, Microbiología y Medicina Preventiva, Hospital Universitario Virgen Macarena/CSIC/Instituto de Biomedicina de Sevilla (IBiS), 41009 Seville, Spain; luiselopezcortes@gmail.com; 4 Infección por el VIH y farmacocinética de antivirals, Instituto de Biomedicina de Sevilla (IBiS), Antonio Maura Montaner Street s/n, 41009 Seville, Spain

**Keywords:** *Enterococcus faecalis*, infective endocarditis, outpatient treatment, outpatient parenteral antibiotic treatment (OPAT), systematic review, treatment alternatives

## Abstract

The selection of the best alternative for *Enterococcus faecalis* infective endocarditis (IE) continuation treatment in the outpatient setting is still challenging. Three databases were searched, reporting antibiotic therapies against *E. faecalis* IE in or suitable for the outpatient setting. Articles the results of which were identified by species and treatment regimen were included. The quality of the studies was assessed accordingly with the study design. Data were extracted and synthesized narratively. In total, 18 studies were included. The treatment regimens reported were classified regarding the main antibiotic used as regimen, based on Aminoglycosides, dual β-lactam, teicoplanin, daptomycin or dalbavancin or oral therapy. The regimens based on aminoglycosides and dual β-lactam combinations are the treatment alternatives which gather more evidence regarding their efficacy. Dual β-lactam is the preferred option for high level aminoglycoside resistance strains, and for to its reduced nephrotoxicity, while its adaptation to the outpatient setting has been poorly documented. Less evidence supports the remaining alternatives, but many of them have been successfully adapted to outpatient care. Teicoplanin and dalbavancin as well as oral therapy seem promising. Our work provides an extensive examination of the potential alternatives to *E. faecalis* IE useful for outpatient care. However, the insufficient evidence hampers the attempt to give a general recommendation.

## 1. Introduction

Infective endocarditis (IE) is a potentially fatal infectious disease, characterized by its elevated morbidity and mortality. In spite of being relative infrequent, it is considered one of the four most common life-threatening infection syndromes [1]. Excluding cases developed among injection drug users, Enterococcal species are the third most common cause of IE, 90–97% of them being produced by *E. faecalis* [1,2]. While IE incidence has remained constant in the last few decades [1], the rate of enterococcal IE has increased along with changes in patients’ characteristics [3,4].

Nowadays, *E. faecalis* IE treatment is still challenging [5]. Enterococcal antimicrobial resistance is a major problem involving not only the inner bacterial resistance mechanisms, but also antibiotic use in the clinical setting and veterinary medicine, which requires a coordinated multidisciplinary approach. *E. faecalis* is a common microorganism in human microbiota, and several protective, but also harmful, roles have been suggested, including a leading role in colorectal cancer [6]. The gold standard treatment for *E. faecalis* IE is a combination therapy with ampicillin plus gentamycin or plus ceftriaxone [1,2]. Nevertheless, the lack of antibiotics with bactericidal effects, the ability for biofilm formation and the increase in antibiotic resistance have hindered the attempts to find a commonly accepted treatment for this endocarditis [1,5,6,7,8]. Moreover, another demanding feature in its management is the length of treatment, for which a minimum of 4–6 weeks is usually recommended [1,2]. These patients experience an initial period which involves a high risk of complications, such as heart failure, perivalvular extension or systemic embolism. Then, patients should remain hospitalized during this period, usually for 10 to 21 days, according to the clinical condition and the infection characteristics [9]. Thereafter, the only reason for remaining hospitalized is to receive intravenous antibiotic therapy [10,11].

At this point, there are several alternatives for outpatient continuation treatment. Oral antibiotic therapy has been explored and strengthened by recent promising results [12,13]. Nevertheless, despite being an attractive alternative that should be contemplated, there is still a lack of solid evidence supporting a specific oral antibiotic regimen for *E. faecalis* IE continuation regimen. Another option in this scenario is outpatient parenteral antibiotic therapy programs (OPAT), which provide an opportunity for discharge, with well-known benefits for both the health-care systems and the patients [11,14]. Recommendations for patient’s requirements and antibiotic selection have been previously published, the latter being determined by the OPAT model and the pharmacokinetic drug’s properties, but also by solution’s stability and safety drug’s profile [11,14,15]. *E. faecalis* IE treatment via OPAT has not been usually recommended [10,14,15,16,17], and sometimes even discouraged [11,18]. Nevertheless, evidence supporting *E. faecalis* IE continuation treatment through OPAT has grown lately, including a variety of antibiotic options, with favorable results [19,20,21,22,23,24,25].

The purpose of this systematic review is to identify, critically appraise and synthesize the evidence from studies reporting different strategies appropriated for outpatient continuation therapy for *E. faecalis* IE.

## 2. Results

### 2.1. Search Results

**Figure 1:**
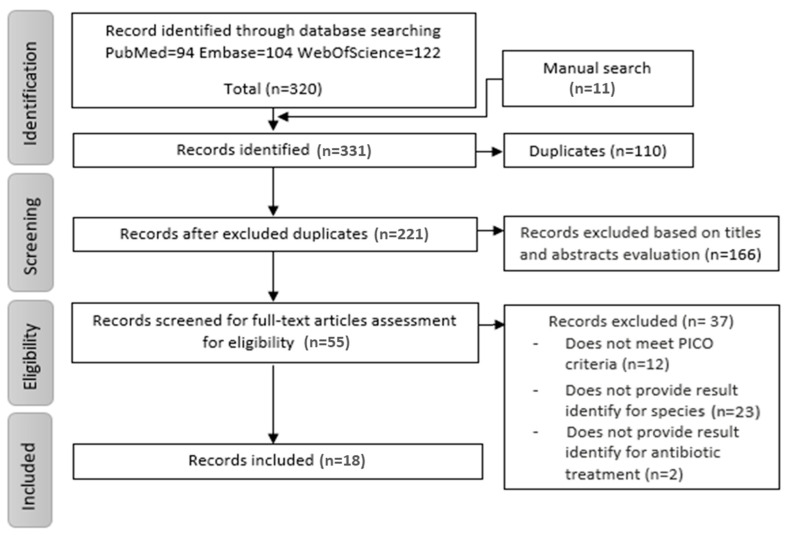
Study selection flowchart.

The chosen search strategy resulted in a total of 320 articles. Additionally, 11 records were identified through reference and citation searching of the included papers. From this selection, we reviewed 55 potentially eligible full-text articles, based on title and abstract evaluation. Following an in-depth reading, a total of 18 articles were finally included in this systematic review (Figure 1).

### 2.2. Overview of the Studies

As shown in Table 1, the study designs of the 18 selected articles included randomized clinical trials (*n* = 1) [12], non-randomized clinical trials (*n* = 1) [26], prospective cohort studies (*n* = 2) [27,28], retrospective cohort studies (*n* = 9) [20,22,23,29,30,31,32,33,34] and case series studies (*n* = 5) [19,21,24,25,35]. Only 10 of them analyzed more than one treatment option [12,20,23,27,28,29,30,31,32,33]. The study clinical settings were outpatient (*n* = 4) [19,24,25,34], inpatient (*n* = 9) [26,27,28,29,30,31,32,33,35] and mixed (*n* = 5) [12,20,21,22,23], and the therapy indication included continuation therapy in nine studies [12,19,20,21,22,23,24,25,34]. The majority of studies included left- and right-sided endocarditis, with the exception of three studies treating only left-sided IE [12,28,33]. There were large variations regarding the follow-up period after the ending of the antimicrobial therapy, ranging between no follow-up [20] and one year after [29,30,33,34]. Mortality was the most common outcome measure, being described in all articles, while relapse incidences were reported in 13 studies [12,19,22,23,24,25,26,27,29,30,31,33,34] and treatment failure in only 11 studies [12,19,20,21,22,25,27,29,30,31,32]. Two articles presented data from the same study population; nevertheless, both were included because different aims and outcomes were assessed [26,27].

### 2.3. Quality of the Studies

The methodological quality of the studies included in this review was variable. Five studies were evaluated as good quality or low risk of bias, 11 studies were assessed as having some concerns or moderate risk of bias and 2 studies were reported as poor quality or serious risk of bias. The detailed results for the risk of bias assessment are summarized in Table 2.

### 2.4. Therapeutic Alternatives

The therapeutic alternatives identified in the articles were grouped into six categories corresponding with the main antibiotic used. The study details are summarized in Table 1.

#### 2.4.1. Aminoglycosides Based Regimens

The clinical outcomes of *E. faecalis* IE patients treated via an initial therapy with a gentamycin-based regimen were evaluated in six studies, comprising 343 episodes, none of which were in OPAT [27,28,29,30,31,33]. The main combination employed was ampicillin 2 g each 4 h plus gentamicin 3 mg/kg/day during 4–6 weeks of treatment, although vancomycin and penicillin G were alternatives to ampicillin in two cases [28,33]. Among the studies, adverse events rates were high, even superior to 40% [27,30,31], mostly due to renal toxicity. On the other hand, relapse and mortality rates oscillated between 3% and 11% and 17% and 35% respectively, after 6–12 months of minimal follow-up. One study limited patient inclusion based on IE type [33]. This study compared the efficacy and safety of short (2 weeks) and long (4–6 weeks) treatment with gentamycin 3 mg/kg/day combined in both cases with 4–6 weeks of ampicillin or penicillin. Renal toxicity observed was significantly lower when the gentamycin short-course was used irrespective of clinical efficacy, which remained similar in both groups (69% vs. 66% of 1 year event-free survival). Likewise, Fernandez-Hidalgo et al. [31] analyzed a large cohort of patients treated with ampicillin plus gentamycin for 4–6 weeks, in which the overall mortality after a median follow-up of 11 months was 25% and the side effects reported were 44%.

#### 2.4.2. Dual β-Lactam Regimens

Nine studies gathered clinical results about 337 *E. faecalis* IE episodes treated with dual β-lactam therapy [19,20,24,25,26,27,29,30,31], three of which were conducted in OPAT, and then collected data from 11 patients [19,24,25]. The follow-up comprised six months (*n* = 6), three months (*n* = 1), and another study referred no follow-up after treatment ending. The most common combination assessed was ampicillin 2 g each 4 h plus ceftriaxone 2 g each 12 h for 4–6 weeks as an initial therapy [26,27,29,30,31]. However, one study [19] adjusted it to their OPAT program, administering ceftriaxone 4 g in single daily dose, and another one [20] administered ampicillin at a dose of 2 g each 6 h. Two studies proffered penicillin G as an alternative to ampicillin in a small cohort (*n* = 7) without any deaths or relapses [24,25]. Embracing all data reported, the mortality rate was between 20% and 30% in four studies [26,27,29,31], and lower than 20% in another four [19,24,25,30]. Only one study reported a mortality rate higher than 30% [20], maybe related to the lower dose of ampicillin administered. Regarding toxicity, the highest adverse events rate was reported by Pericas et al. [27], showing 34% of renal failure in the cohort, although only 3% of discontinuation due to toxicity. Suzuki et al. [24] described adverse effects in one of the four patients reported (25%), while the percentage of adverse events reported was lower than 20% in the other seven studies [19,20,25,26,29,30,31]. In the largest cohort evaluated in the field [31] (*n* = 159), the overall mortality rate was 26%, along with a low adverse events rate (9%), despite poorer general conditions than in the comparator group. Across the studies, the main conclusion was that dual β-lactam therapy with ampicillin is a primary option for *E. faecalis* IE, while penicillin G could be an alternative.

#### 2.4.3. Teicoplanin-Based Regimens

A total of 56 patients treated with teicoplanin were reported [22,23,35], most of them (89%) as continuation or salvage therapy. Two studies compromised episodes treated in OPAT [22,23]. The largest study conducted embraced the use of monotherapy with teicoplanin for treating *E. faecalis* IE as continuation therapy. The reported mortality related to IE was low (8%), but the population treated with teicoplanin suffered from less severe IE than the standard therapy group [23].

Overall, the dose regimens were highly variable among the studies. Two of them introduced a loading dose, but the mean maintenance dose varied between 5.8 and 10 mg/kg/day [22,23], while in the other one, fixed doses were used [35]. Within the patients treated with teicoplanin as a continuation or salvage therapy, 16 died (32%) in a minimal follow-up period of 3 months. Only three relapses were reported in these studies. The comprehensive conclusion of the studies was that teicoplanin could be an alternative for the sequential treatment of this syndrome.

#### 2.4.4. Daptomycin Based Regimens

*E. faecalis* IE treatment with daptomycin was assessed in 3 studies, including 26 patients [20,28,32]. The treatment scheme was considerably heterogeneous, included initial and salvage therapy, monotherapy and combine regimens, and the mean doses ranged between 8.5 and 10.125 mg/kg/day. Mortality rates reported were low (0–22%), although only one study [28] attained more than a one-month follow-up. Ceron et al. [20] included OPAT treatment and described the salvage treatment of 5 *E. faecalis* IE episodes, of which four needed a treatment change due to treatment failure. So, the stated final conclusions differed, with two supporting daptomycin as an alternative treatment in this scenario [28,32], and one showing some concerns [20].

#### 2.4.5. Dalbavancin Regimens

Two articles [21,34] disclosed the outcomes of seven *E. faecalis* IE patients treated with dalbavancin, six (86%) with OPAT. The dosage regimen and length of the therapy encompassed single and multiple variable doses for 1 to more than 6 weeks. In these cohorts, six patients were successfully treated with dalbavancin in OPAT, hence it was proposed as an alternative.

#### 2.4.6. Oral Therapy

Oral therapy has been evaluated in one large randomized clinical trial [12]. This study comprised 400 left-side IE episodes, but it should be noted that only 20% of patients screened were included. Among them, continuation treatment with outpatient oral therapy and inpatient intravenous therapy were settled on in 201 and 199 patients, respectively. In total, 51 *E. faecalis* left-side IE episodes were enclosed in the oral arm. The mean time from diagnosis until the beginning of continuation of oral therapy was 17 days. High variability was shown among the antibiotic selection against *E. faecalis*, with six different regimen options used. Amoxicillin was part of the treatment in 90% (46/51) of these oral regimens given. The primary outcome was a composite endpoint that encompassed all-cause mortality, unplanned cardiac surgery, clinically evident embolic events or relapse of bacteremia. Considering patients with IE caused by E. faecalis treated with oral therapy, it occurred in four (7.8%), comprising only one death (1.9%).

## 3. Discussion

This review included treatment alternatives for *E. faecalis* IE suitable for the outpatient setting, including oral or OPAT treatment. To this end, little evidence was found to support optimal continuation treatment. In fact, outpatient treatment of these patients has been a matter of discussion, and there is no consensus regarding patient and treatment selection [9]. To the best of our knowledge, there is no previous work summarizing the current evidence related to *E. faecalis* IE treatment appropriate for outpatient care. The two main findings were the lack of randomized clinical trials and large observational studies, and the heterogeneity regarding methodology and follow-up period among the included studies. Despite this, the alternatives analyzed in this review provided a wide view on the outpatient continuation treatment for this infection.

On the one hand, the use of aminoglycosides to synergize with cell wall-active agents in the initial treatment of *E. faecalis* IE has been the first choice treatment for decades, in defiance of the high rates of nephrotoxicity observed [1,2]. Lately, doubts about its clinical utility have been raised [36]. This review included six studies in which this treatment option has been used as standard therapy [27,28,29,30,31,33]. That said, the toxicity of prolonged treatment, and the subsequent need for drug monitoring, have been obstacles for OPAT incorporation [10]. In this sense, a combination regimen based on a reduced gentamicin course interesting for OPAT purposes was previously proposed [37] and thereafter deeply studied [33]. They found a greater decrease in nephrotoxicity compared to the long gentamicin course, without repercussions in efficacy rates. The reduction in toxicity and regimen complexity are advantageous properties for OPAT inclusion. However, none of these studies have been carried out in OPAT, and also these results are only suitable for non-high-level aminoglycoside resistance (non-HLAR) strains, so its applicability could be reduced.

On the other hand, dual β-lactam therapy has been raised as a noteworthy alternative for initial treatment in the field. The safety and efficacy of this combination was substantiated in a non-randomized clinical trial [26] and a large prospective observational study [31], followed by several retrospective cohort studies that confirm their findings [27,29,30]. Despite there being enough evidence to support the use of the dual β-lactam combination in inpatient treatments, its adaptation to OPAT is complex and has been poorly studied.

Several options should be considered. The first one could be to continue using the intra-hospital regimen of ampicillin 2 g each 4 h administered through an electronic pump plus ceftriaxone 2 g each 12 h. In order to avoid the unfeasible use of two pumps simultaneously, this regimen would require the continuous assistance of a caregiver, or patient autonomy for venous access manipulation. That option is not suitable for all types of OPAT organizations, and it would remarkably reduce the patient candidates for it. Similar difficulties are found in the alternative dual β-lactam regimen [24,25], where ampicillin has been replaced by penicillin G due to discrepancies in ampicillin solution stability. Moreover, the small population studied and the lack of synergistic activity studies for the penicillin G plus ceftriaxone combination lessen its utility. For a long time, ampicillin stability data were contradictory, whereas recent studies have solved the disagreement [38,39,40]. Besides, ampicillin and ceftriaxone daily doses diluted in the same solution were found to be stable, which could be considered as an alternative to administration through a single pump [40]. Another suggested alternative is to switch the ceftriaxone dose regimen to 4 g in single daily doses. Four patients were treated with this combination with successful outcomes [19]. Nonetheless, together with the small number of patients included, a serious concern about this modification is that optimal ceftriaxone exposure to achieve synergistic concentrations with ampicillin has not been described [41,42]. Altogether, dual β-lactam adaptation to OPAT warrants further investigation for the establishment of an optimal regimen.

In the last few years, three new intravenous alternatives have emerged. Firstly, daptomycin could be an alternative in this scenario. Among and within the studies assessing daptomycin efficacy in IE episodes, noteworthy heterogeneity was found in the therapeutic approach, but also in the conclusion reported [20,28,32]. The poor results stated by Ceron et al. [20] could dismiss the daptomycin recommendation supported by the promising results reported in other studies [28,32]. Nevertheless, it should be noted that this study includes a small population and the data regarding patients switching between treatment groups are unclear.

Teicoplanin is another antibiotic possible alternative in this situation. It is a popular agent for OPAT owing to its long elimination half-life that enables once-daily dosing as well as intramuscular or subcutaneous administration, which could preclude permanent vascular access. The studies assessing the effectiveness of this alternative, which encompassed outpatient treatments, have shown promising outcomes [22,23,35]. Nevertheless, the proposed dose regimen highly varies with respect to dose (5–10 mg/kg/day), and also loading dose use and length of therapy. As it currently stands, data providing support for the optimal dose and route of administration for teicoplanin options remain uncertain. Finally, Gram-positive IE treatment with dalbavancin has been studied [21,34], and included *E. faecalis* IE. However, the regimen design was inconsistent and the *E. faecalis* sample size small. Dalbavancin is a welcome agent in OPAT programs due to its extended dosing interval and the reduction of health-care visit needed, although it is not accessible worldwide due to its high cost. Nevertheless, for hospitals without OPAT programs, it might be a cost-effective alternative. In conclusion, any of these alternatives should be considered for the continuation treatment of *E. faecalis* IE, but further research is needed to analyze the efficacy and safety in larger cohorts, and to standardize the optimal dose regimen. These two last options attract particular attention due to the inner benefits, previous experience in the outpatient use and management, and, in the case of teicoplanin, the number of successfully treated patients. Thus, they might be considered the two more reasonable intravenous alternatives for continuation treatment for *E. faecalis* IE patients.

Lastly, partial oral treatment for IE has been assessed [12]. The authors demonstrated that switching to oral therapy was non-inferior to continued intravenous therapy after initial intravenous treatment. From their results, amoxicillin-based regimens emerge as a treatment option in this field. Nonetheless, oral therapy entails normal gastrointestinal uptake and good adherence, ensured in non-self-administered OPAT. Antibiotic exposure in high inoculum infections after oral antibiotic administration depends on the pharmacokinetics profile of each drug, and nowadays it is a matter of discussion, especially regarding β-lactam oral therapy [13,43,44]. In this trial, seven patients in the oral therapy arm showed antibiotic plasma concentrations lower than the effective level, whereas no regimen adjustment was made on that base [12]. Linezolid, an appealing option due to its bioavailability, has shown conflicting results [44,45,46], thus its use for IE treatment is not systematically recommended [1,2]. Despite being an encouraging option, oral antibiotic therapy warrants further investigation to elucidate the best drug choice and regimen in each scenario, and also for patient selection.

The present review has a number of limitations and strengths. Observational studies were included due to the historical difficulties of performing randomized controlled trials in IE. Nonetheless, the study has been performed following PRISMA recommendation, and included an in-depth assessment of the studies’ quality. For most of the alternatives discussed, inconsistent regimen designs and doses have been proposed. As such, in spite of the inclusion of a large enough population for drawing conclusions, in some of them, is not possible to recommend a precise regimen. Another limitation was the exclusion of studies based on the lack of information determined by microorganism or by treatment alternative, and the limited data available about actual OPAT IE treatment. The extrapolation of data from the inpatient to the outpatient setting could be inaccurate due to big differences in drug delivery and monitoring. Furthermore, incomplete retrieval is possible for papers outside the databases searched. Onn the other hand, we have conducted a rigorous search and systematic review accompanied by a narrative synthesis. Although previous work summarizing *E. faecalis* infective endocarditis treatment has been conducted lately [5,47], there is an absence of previous reviews gathering evidence concerning outpatient alternatives and possible adaptations of the current treatments, which highlights the novelty and relevance of this review. Our work focused on treatment alternatives suitable for outpatient treatment and early discharge options, which are of great clinical and scientific interest. Our analysis provides a sense of what alternatives could safely be used in this setting, and which alternatives are promising but require further research. It also provides a unique and valuable contribution to the available literature.

## 4. Materials and Methods

Our review protocol was prospectively registered on the PROSPERO international prospective register of systematic reviews (Protocol ID:154593) and is reported in accordance with the Preferred Reporting Items for Systematic Reviews and Meta-Analyses (PRISMA) statement [48].

### 4.1. Search Strategy

**Figure 2:**
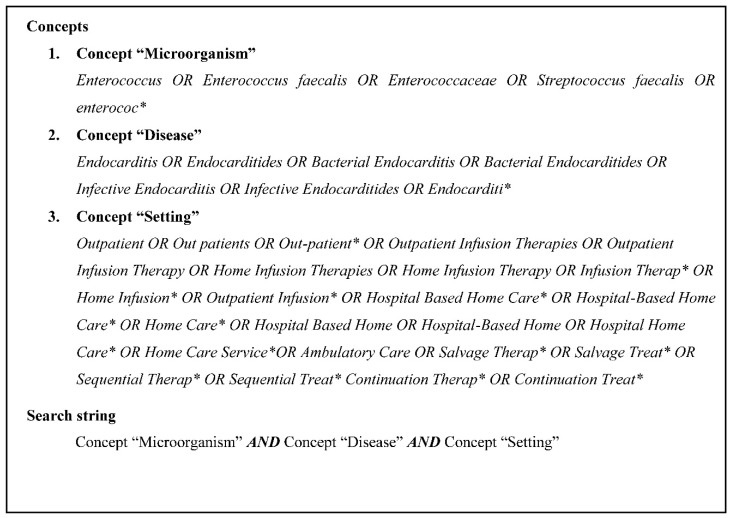
Search strategy.

Our systematic search strategy was developed to seize all articles related to appropriate *E. faecalis* IE antimicrobial treatment in the outpatient setting. We conducted a search in the MEDLINE (through PubMed interface), EMBASE and Web of Science Core collection databases from inception until October 2019, using MeSH terms and keywords associated with the concepts presented in Figure 2. The search strategy was amended according to the functionality of each of the databases. In addition, articles of interest identified by citation tracing were included.

### 4.2. Eligibility Criteria

The inclusion of cohort studies and series of cases in a systematic review has been discouraged [49], however the lack of better evidence in the field forced us to include all types of human studies except case reports. Studies were eligible for review if they were published in English or Spanish and reported clinical outcomes from *E. faecalis* IE patients treated as inpatient or outpatient with oral or intravenous antibiotics appropriated for continuation therapy. We allowed the inclusion of studies reporting data from inpatient treatments where the antibiotics used were included in OPAT guidelines [11,14], or their use in OPAT was already reported, and considered them as “suitable for OPAT”. Likewise, we included studies which partially included information not identified by microorganism or antibiotic regimen, but this data was dismissed. The eligibility criteria applied in this study are presented in detail in Table 3.

### 4.3. Selection of Studies

All the titles and abstracts of the citations identified by our database and manual search were screened after duplicate removal. Relevant articles or those with insufficient information within the title and abstract were full-text assessed. This selection was independently performed by two reviewers (LHH and MVGN) and, in case of uncertainty, discussed until consensus was reached.

### 4.4. Quality Assessment

To evaluate the quality of the studies selected for inclusion, we used three tools according to the study design: A Cochrane risk-of-bias tool for randomized trials (ROB.2) was used for randomized controlled trials [50] and the Risk of Bias in Non-Randomized Studies of Interventions tool (ROBINS-I) was suitable for non-randomized studies [51]. For case series studies, quality was assessed using a standardized Study Quality Assessment Tool (SQAT) designed by the National Heart, Lung, and Blood Institute under the National Institutes of Health [52]. When using the ROBINS-I tool the overall risk of bias of the paper was categorized into “Low”, “Moderate”, “Serious” or “Critical”. When ROB.2 was applied, risk of bias was classified into the “Low”, “High” or “Some concerns” categories. Finally, when using the NIH Quality Assessment Tool, the reported risk of bias was summarized as “Good”, “Fair” or “Poor”.

### 4.5. Data Extraction and Synthesis

Data extraction was performed by one reviewer (LHH) using a standardized data extraction form; any uncertainty was discussed with another investigator (MVGN). We extracted the following information from the studies: Study design, treatment setting (outpatient or inpatient), type of endocarditis (left- or right-side endocarditis, native or prosthetic valve endocarditis or cardiac device-related endocarditis), endocarditis definition, follow-up period, treatment alternative (antibiotic or antibiotic combination, dose regimen and route of administration), number of *E. faecalis* IE patients treated, adverse events, clinical outcomes (mortality, relapses and others) and key findings. Given the heterogeneity of the study designs, interventions and outcome measures, it was not feasible to pool the results in a meta-analysis. Alternatively, we performed a narrative synthesis of evidence following the Cochrane Consumers and Communication Review Group’s guidelines [53]. Studies were grouped according to the antibiotic alternative reported, individual study characteristics and findings were summarized, and similarities, differences and patterns were identified.

## 5. Conclusions

In this review, the therapeutic treatments against *E. faecalis* were reviewed, with special focus on outpatient therapy. The best option for continuation outpatient therapy after discharge is still unknown. The gold standard options for inpatient treatment require regimen adjustments which are poorly studied. New attractive alternatives for OPAT are arising, especially teicoplanin and dalbavancin regimens, the pharmacokinetics profiles and ease of administration of which provide significant advantages for outpatient treatment, whereas their safety and efficacy are not strongly evidence-supported yet. The assessment of the safety and efficacy of the suggested alternatives against *E. faecalis* IE in outpatient warrants future investigations.

## Figures and Tables

**Table 1:** Summary of the results.

Ref	Study Design/Setting	Endocarditis Type and Definition	Follow-Up Period	Dose Regimen	EFIE/Total Patients	Surgical Treatment	Adverse Events	Clinical Outcomes			Key Finding
								Mortality	Relapses	Others	
**Aminoglycoside based regimen**											
[28]	Prospective cohort study with comparator/Inpatient	LS-NVE 28/43 LS-PVE 15/43 Modified Duke criteria	6 months	Initial therapy A/V plus G	43/149	26/43 (60.5%)	ND	Overall 15/43 (35%) In hospital 6/43 (14%)	ND	Days of bacteriemia 3.0 (1.5–5.0) ^b^	High-dose daptomycin may be a valid alternative to standard therapy for left-side *E. faecalis* IE
[29]	Retrospective cohort study with comparator/Inpatient	NVE 9/9 Modified Duke criteria	1 year	Initial therapy A 2 g/4 h plus G 3 mg/kg/day for 4 weeks	9/9	2/9 (22.2%)	2/9 (22%)	1-year 3/9 (33%) In hospital 3/9 (33%)	1/9 (11%)	Discontinuation of AB therapy 2/9 (22%)	The suitability of a short course of antibiotic treatment for uncomplicated *E. faecalis* IE should be readdressed.
		NVE 14/23 PVE 9/23 Modified Duke criteria		Initial therapy A 2 g/4 h plus G 3 mg/kg/day for 6 weeks	23/23	14/23 (60.8%)	2/23 (8%)	1-year 7/23 (30%) In hospital 6/23 (26%)	1/23 (4%)	Discontinuation of AB therapy 9/23 (39.1%)	
[33]	Retrospective cohort study with comparator/Inpatient	LSE ND	1 year	Initial therapy PG/A for 4–6 weeks plus G 3 mg/kg/day for 2 weeks	43/43	15/43	eGFR change −1 (−13 to 4) ^b^ mL/min	In hospital 2/43 (5%)	2/43 (5%)	1 year evento-free survival 27/43 (69%)	G treatment for 2 weeks, rather than 4–6 weeks, seems adequate and preferable in susceptible *E. faecalis* IE
				Initial therapy PG/A plus G 3 mg/kg/day for 4–6 weeks	41/41	14/41 (34.1%)	eGFR change −11(−25 to−3) ^b^ mL/min	In hospital 4/41 (10%)	3/41 (7%)	1 year evento-free survival 27/41 (66%)	
[30]	Retrospective cohort study with comparator/Inpatient	All types (ND) Modified Duke criteria	1 year	Initial therapy A 2 g/4 h plus G 3 mg/kg/day for 4–6 weeks	67/67	24/67 (35.8%)	20/67 (66%)	1-year 11/67 (17%) 3 months 9/67 (14%)	1/67 (3%)	Fail to complete therapy 30/67 (49%)	AC is a safe alternative to AG for treating *E. faecalis* IE
[27]	Retrospective cohort study with comparator/Inpatient	NVE 20/30 PVE 9/30 CDRE 1/30 Modified Duke criteria	392 (118.5–792.0) ^b^ days	Initial therapy A 2 g/4 h plus G 3 mg/kg/day for 4–6 weeks	30/30	15/30 (50%)	Renal failure 19/30 (64%)	1-year 9/30 (30%) In hospital 8/30 (27%)	2/30 (3%)	Discontinuation due to toxicity 13/30 (43%)	The efficacy of 6 weeks treatment with AC appears similar and safer than 4–6 weeks treatment with AG
[31]	Prospective cohort study with comparator/Inpatient	NVE 57/87 PVE 30/87 Modified Duke criteria	11.1 (4.4–22.5) ^b^ months	Initial therapy A 2 g/4 h plus G 3 mg/kg/day for 4–6 weeks	87/87	35/87 (40.2%)	38/87 (44%)	Overall 22/87 (25%) During treatment 8/87 (21%)	3/87 (3.4%)	Treatment change 2/87 (2%)	AC combination was as effective as AG, with less adverse events.
**Dual β-lactam regimens**											
[20]	Retrospective cohort study with comparator/Inpatient and outpatient	LS-NVE 10/21 LS-PVE 9/21 RSE 2/21 Modified Duke criteria	During antibiotic therapy	Initial treatment A 2 g/6 h + C 2 g/12 h	21/21	4/21 (19%)	0%	9/21 (43%)	ND	Treatment change 0/21 (0%) Days of bacteraemia 1 (1–6) ^b^	Daptomycin treatment for enterococcal endocarditis lead to worse outcomes than AC therapy
[19]	Case series study without comparator/Outpatient	LS-NVE 4/4 ND	365 (221–406) ^b^ days	Continuation therapy A 2 g/4 h plus C 4 g/24 h for 6 weeks antibiotic therapy	4/4	3/4(75%)	0/4(0%)	0/4 (0%)	0/4(0%)	Treatment change 0/4(0%)	A high single daily dose of C plus A could be an option as a continuation therapy in an OPAT program for *E. faecalis* IE.
[29]	Retrospective cohort study with comparator/Inpatient	NVE 14/14 Modified Duke criteria	1 year	Initial therapy A 2 g/4 h plus C 2 g/12 h for 4 weeks	14/14	3/14 (21.4%)	2/14 (14%)	1-year 3/14 (21%) In hospital 2/14 (14%)	2/14 (14%)	Discontinuation of AB therapy 1/14 (7.1%)	The suitability of a short course of antibiotic treatment for uncomplicated EFIE should be readdressed.
		NVE 14/32 PVE 18/32 Modified Duke criteria		Initial therapy A 2 g/4 h plus C 2 g/12 h for 6 weeks	32/32	14/32 (43.7%)	1/32 (3%)	1-year 8/32 (25%) In hospital 8/32 (25%)	0/32 (0%)	Discontinuation of AB therapy 1/32 (3.1%)	
[30]	Retrospective cohort study with comparator/Inpatient	All types (ND) Modified Duke criteria	1 year	Initial therapy A 2 g/4 h plus C 2 g/12 h for 4–6 weeks	18/18	3/18 (16.6%)	1/18 (20%)	1-year 3/18 (17%) 3 months 3/18 (17%)	1/18 (14%)	Fail to complete therapy 5/18 (28%)	AC is a safe alternative to AG for treating *E. faecalis* IE
[27]	Retrospective cohort study with comparator/Inpatient	NVE 25/39 PVE 13/39 CDRE 1/39 Modified Duke criteria	392 (118.5–792.0) ^b^ days	Initial therapy A 2 g/4 h plus C 2 g/12 h for 4–6 weeks	39/39	15/39 (39%)	Renal failure 13/39 (34%)	In-hospital 9/39 (23%) 1-year 10/39 (26%)	3/39 (8%)	Discontinuation due to toxicity 1/39 (3%)	The efficacy of 6 weeks treatment with AC appears similar and safer than 4–6 weeks treatment with AG
[31]	Prospective cohort study with comparator/Inpatient	NVE 98/159 PVE 59/159 CDRE 2/159 Modified Duke criteria	11.1 (4.4–22.5) ^b^ months	Initial therapy A 2 g/4 h plus C 2 g/12 h for 4–6 weeks	159/159	53/159 (33.3%)	14/159 (9%)	Overall 42/159 (26%) During treatment 35/159 (22%)	3/159 (1.8%)	Treatment change 2/159 (1%)	AC combination was as effective as AG, with less adverse events.
[26]	Non randomized clinical trial without comparator/Inpatient	All types(ND) Modified Duke criteria	3 months	Initial therapy A 2 g/4 h plus C 2 g/12 h for 42 (5–48)^b^ days	43/43	7/43 (16.3%)	2/43 (4.6%)	Overall 12/43 (28%) During treatment 10/43 (23%)	2/43 (4.6%)	ND	AC may be a treatment option for *E. faecalis* endocarditis
[24]	Case series study without comparator/Outpatient	NVE 3/4 PVE 1/4 Modified Duke criteria	6 months	Continuation therapy PG 24 million U PC/24 h plus C 2 g/12 h for 6–8 weeks antibiotic treatment	4/4	0/4 (0%)	1/4 (25%)	0/4 (0%)	0/4 (0%)	ND	PG plus C would be effective in the treatment of *E. faecalis* IE
[25]	Case series study without comparator/Outpatient	ND	3 months	PG 18–24 million U PC/24 h plus C 2 g/12 h for 6 weeks antibiotic therapy	3/3	ND	0/3 (0%)	0/3 (0%)	0/3 (0%)	Treatment change 0/3 (0%)	PG plus C maybe an alternative for the treatment of *E. faecalis* IE
**Teicoplanin based regimens**											
[22]	Retrospective cohort study without comparator/ Inpatient and outpatient	NVE 16/22 PVE 5/22 Non valvular E 1/22 Modified Duke criteria	3 months	First- line (1/14) Salvage therapy (13/14) LD + 10 (10–10.8) ^b^ mg/kg/day for 43.5 (38.8–56.3) days ^b^	14/22	3/14 (21.4%)	2/14 (14%)	During treatment 1/14 (7.1%) 3 months 2/14 (14%)	0/14 (0%)	Treatment change 2/14 (14%)	Teicoplanin can be used in *E. faecalis* IE as a sequential treatment
[23]	Retrospective cohort study with comparator/Inpatient and outpatient	NVE 21/37 PVE 16/37 Modified Duke criteria	783 (126–1227) ^b^ days	Continuation therapy LD + 5.8 mg/kg/day 39 (25–34) ^b^ days antibiotic therapy	37/37	11/37 (30%)	ND	Global 14/37 (38%) IE-related 3/37 (8%)	3/37 (8%)	Patients who did not die from *E. faecalis* IE or experience relapses 33/37 (89%)	Teicoplanin sequential treatment appears to be effective in selected patients
[35]	Case series study without comparator/Inpatient	All type ND	6 months	Initial therapy 600 mg/day for 5–6 weeks	5/26	ND	ND	0/5 (0%)	ND	ND	Teicoplanin initial treatment was effective for *E. faecalis* IE
**Daptomycin based regimens**											
[20]	Retrospective cohort study with comparator/Inpatient and outpatient	LS-NVE 4/6 LS-PVE 1/6 CDRE 1/6 Modified Duke criteria	During antibiotic therapy	Initial therapy (1/5) Salvage (4/5) 8.5 (6–10) mg/kg/day	5/6	3/6 (50%)	0%	1/6 (16.7%)	ND	Treatment change 4/5 (80%) ^a^ Days of bacteriemia 6 (1–13) ^b^	Daptomycin treatment for enterococcal endocarditis lead to worse outcomes than AC therapy
[28]	Prospective cohort study with comparator/Inpatient	LS-NVE 7/9 RS-NVE 2/9 Modified Duke criteria	6 months	Initial (8/9) or salvage (1/9) 8.3 (7.1–9.4) ^b^ mg/kg for 28.5 (22.0–42.5)^b^ alone or in combination	9/29	4/9 (44.4%)	0/9 (0%)	Overall 2/9 (22%) In-hospital 1/9 (11.1%)	ND	Days of bacteriemia 2.0 (1.5–3.0) ^b^	High-dose daptomycin may be a valid alternative to standard therapy for left-side *E. faecalis* IE
[32]	Retrospective cohort study with comparator/Inpatient	NVE 8/12 PVE 4/12 Modified Duke criteria	30 days	First-line or salvage therapy with daptomycin-based regimen 10.125 (8–12) ^b^ mg/kg for 45 ± 21.1 days	12/16	7/16 (43.7%)	ND	30-days 0/12 (0%)	2/12 (0%)	Treatment failure 0/12 (0%)	Daptomycin could be an alternative treatment option for enterococcal NVE and PVE.
**Dalbavancin based regimen**											
[21]	Case series study without comparator/Inpatient and outpatient	NVE 3/4 PVE 1/4 Modified Duke criteria	6 months	Initial, salvage or continuation therapy LD (1000–1500 mg) plus 500–1000 mg once or twice weekly for 1 to >6 weeks	4/27	ND	0/4 (0%)	1/4 (25%)	ND	Treatment failure 1/4 (25%)	Dalbavancin is effective and safe for prolonged treatment.
[34]	Retrospective cohort study without comparator/Outpatient	NVE 2/3 PVE 1/3 Modified Duke criteria	1 year	Continuation therapy 500–1500 mg between 1 and 4 doses	3/34	12/34	0/3	0/3	0/3	Cure of infection 3/3	Dalbavancin is an effective consolidation antibiotic therapy in clinically stabilized patients with IE
**Oral therapy**											
[12]	Randomized clinical trial with comparator/Outpatient	LSE Modified Duke criteria	6 months	Continuation therapy with oral antibiotics with amoxicillin alone or plus moxifloxacin/linezolid/rifampicin/ciprofloxacin or moxifloxacin plus linezolid for 17 (14–25) days ^b^	51/201	15/51 (29.4%)	10/201 (5%)	All-cause 1/51 (1.9%)	3/51 (5.8%)	AB route change 4/201 (1.9%) Composite endpoint ^c^ 4/51 (7.8%)	Continuation therapy with oral antibiotics is non-inferior than intravenous therapy

Legend—AB: Antibiotic. Ref: Reference. LD: Loading dose. A: Ampicillin. C: Ceftriaxone. G: Gentamycin. PG: Penicillin G. V: Vancomycin. All-type endocarditis included left-side (LSE) and right-side endocarditis (RSE), native (NVE) or prosthetic (PVE) valve endocarditis and cardiac device-related endocarditis (CDRE). ND: No data. ^a^ All changes in the daptomycin group were due to treatment failure. ^b^ [median (range)]. ^c^ Composite endpoint: all-cause mortality, unplanned cardiac surgery, clinically evident embolic events, or relapse of bacteremia.

**Table 2:** Quality assessment of the studies.

**Study**		**Risk of Bias Due To**									
	**Tool**	**Confounding**	**Selection of Participants**	**Classification of Interventions**		**Deviations from Intended Interventions**	**Missing Data**	**Measurement of Outcomes**		**Selection of the Reported Result**	**Overall Bias**
[20]	Robins	S	L	L		M	L	M		M	S
[21]		S	L	L		S	M	M		L	S
[22]		M	L	L		L	L	L		L	M
[23]		M	L	L		L	M	L		M	M
[29]		M	L	L		NI	L	M		M	M
[33]		M	L	L		L	L	L		L	M
[28]		M	L	L		L	NI	L		L	M
[30]		M	L	L		M	L	L		L	M
[27]		M	L	L		M	L	L		L	M
[26]		L	L	L		NI	L	L		L	L
[31]		M	L	L		M	L	L		L	M
[32]		M	L	M		NI	NI	L		L	M
[34]		M	L	M		NI	L	L		L	M
**Study**	**Tool**	**Randomization Process**		**Deviations from Intended Interventions**			**Missing Data**	**Measurement of Outcomes**		**Selection of the Reported Result**	**Overall Bias**
[12]	ROB-2	L		SC			L	L		SC	SC
**Study**	**Tool**	**Study Question**	**Population**	**Consecutive**	**Cases Comparable**	**Intervention**	**Measurement of Outcomes**	**Length of Follow Up**	**Statistical Methods**	**Results**	**Overall**
[19]	SQAT	Y	Y	Y	Y	Y	Y	Y	NA	Y	GOOD
[35]		Y	Y	NR	Y	Y	Y	Y	NR	Y	GOOD
[24]		Y	Y	Y	Y	Y	Y	Y	NA	Y	GOOD
[25]		Y	Y	Y	Y	Y	N	Y	NA	N	POOR

Legend: L = Low/M = Moderate/S = Serious/SC = Some concerns/Y = YES/N = NO/NA = Not applicable/NR = Not reported/NI= Not informed.

**Table 3:** Eligibility criteria.

	Inclusion Criteria	Exclusion Criteria
Design	Randomized controlled trials, non-randomized trials and observational studies	Case report, in vivo studies, in vitro studies and non-primary sources
Population	Patients suffering from *E. faecalis* IE	Non-human studies
Intervention/comparator	Antibiotic treatment alternatives	Non-medical approaches (e.g., Surgery)
Context	Outpatient setting * or continuation treatment	-
Outcome	Mortality, relapses, clinical cure, microbiological cure	Any outcome identify by antibiotic treatment and causative microorganism

* Regimens suitable for outpatient setting were included.

## References

[1] Baddour L.M., Wilson W.R., Bayer A.S., Fowler V., Tleyjeh I.M., Rybak M.J., Barsic B., Lockhart P.B., Gewitz M.H., Levison M.E. (2015). Infective Endocarditis in Adults: Diagnosis, Antimicrobial Therapy, and Management of Complications. Circulation.

[2] Habib G., Lancellotti P., Antunes M.J., Bongiorni M.G., Casalta J.-P., Del Zotti F., Dulgheru R., El Khoury G., Erba P.A., Iung B. (2015). 2015 ESC Guidelines for the management of infective endocarditis. Eur. Heart J..

[3] Olmos C., Vilacosta I., Fernandez-Perez C., Bernal J.L., Ferrera C., García-Arribas D., Pérez-García C.N., Román J.A.S., Maroto L., Macaya C. (2017). The Evolving Nature of Infective Endocarditis in Spain. J. Am. Coll. Cardiol..

[4] Fernández-Hidalgo N., Mas P.T. (2013). Epidemiology of Infective Endocarditis in Spain in the Last 20 Years. Rev. Española Cardiol. (Engl. Ed.).

[5] Beganovic M., Luther M.K., Rice L.B., Arias C.A., Rybak M.J., Laplante K.L. (2018). A Review of Combination Antimicrobial Therapy for Enterococcus faecalis Bloodstream Infections and Infective Endocarditis. Clin. Infect. Dis..

[6] García-Solache M., Rice L.B. (2019). The Enterococcus: A Model of Adaptability to Its Environment. Clin. Microbiol. Rev..

[7] Rosa R., Creti R., Venditti M., D’Amelio R., Arciola C.R., Montanaro L., Baldassarri L. (2006). Relationship between biofilm formation, the enterococcal surface protein (Esp) and gelatinase in clinical isolates of Enterococcus faecalis and Enterococcus faecium. FEMS Microbiol. Lett..

[8] Cercenado E. (2011). Enterococcus: Resistencias fenotípicas y genotípicas y epidemiología en España. Enferm. Infecc. Microbiol. Clin..

[9] Pericà J.M., Llopis J., González-Ramallo V., Goenaga M.Á., Muñoz P., García-Leoni M.E., Fariñas M.C., Pajarón M., Ambrosioni J., Luque R. (2019). Outpatient Parenteral Antibiotic Treatment for Infective Endocarditis: A Prospective Cohort Study From the GAMES Cohort. Clin. Infect. Dis..

[10] Shah A.B., Norris A.H. (2016). Handbook of Outpatient Parenteral Antimicrobial Therapy for Infectious Diseases.

[11] López-Azkarreta Í., Martínez A.M., De Mandojana M.F.M., Martín N., Gil Bermejo M., Aznar J.S., Bruguera E.V., Cantero M.J.P., Gentil P.R., Vicente M.D. (2019). Executive summary of outpatient parenteral antimicrobial therapy: Guidelines of the Spanish Society of Clinical Microbiology and Infectious Diseases and the Spanish Domiciliary Hospitalisation Society. Enferm. Infecc. Microbiol. Clin..

[12] Iversen K., Ihlemann N., Gill S.U., Madsen T., Elming H., Jensen K.T., Bruun N.E., Høfsten D.E., Fursted K., Christensen J.J. (2019). Partial Oral versus Intravenous Antibiotic Treatment of Endocarditis. N. Engl. J. Med..

[13] Brown E., Gould F.K. (2020). Oral antibiotics for infective endocarditis: A clinical review. J. Antimicrob. Chemother..

[14] Norris A.H., Shrestha N., Allison G.M., Keller S.C., Bhavan K.P., Zurlo J.J., Hersh A.L., A Gorski L., A Bosso J., Rathore M.H. (2018). 2018 Infectious Diseases Society of America Clinical Practice Guideline for the Management of Outpatient Parenteral Antimicrobial Therapya. Clin. Infect. Dis..

[15] Chapman A.L.N., Seaton R.A., Cooper M.A., Hedderwick S., Goodall V., Reed C., Sanderson F., Nathwani D. (2012). On behalf of the BSAC/BIA OPAT Project Good Practice Recommendations Working Group Good practice recommendations for outpatient parenteral antimicrobial therapy (OPAT) in adults in the UK: A consensus statement. J. Antimicrob. Chemother..

[16] Andrews M.-M., Von Reyn C.F. (2001). Patient Selection Criteria and Management Guidelines for Outpatient Parenteral Antibiotic Therapy for Native Valve Infective Endocarditis. Clin. Infect. Dis..

[17] Rehm S.J. (1998). Outpatient Intravenous Antibiotic Therapy for Endocarditis. Infect. Dis. Clin. N. Am..

[18] Oliveira P.R., Carvalho V.C., Cimerman S., Lima A.L.L.M. (2017). Recommendations for outpatient parenteral antimicrobial therapy in Brazil. Braz. J. Infect. Dis..

[19] Gil-Navarro M.V., López-Azkarreta Í., Luque-Marquez R., Gálvez J., De Alarcon-Gonzalez A. (2017). Outpatient parenteral antimicrobial therapy inEnterococcus faecalisinfective endocarditis. J. Clin. Pharm. Ther..

[20] Cerón I., Bermejo J., Bouza E., Eworo A., Cruz A.F., Cuerpo G., Robles J.A.G., Vecchio M.G.-D., Mansilla A.G., González-Ramallo V. (2014). Efficacy of daptomycin in the treatment of enterococcal endocarditis: A 5 year comparison with conventional therapy. J. Antimicrob. Chemother..

[21] Tobudic S., Forstner C., Burgmann H., Lagler H., Ramharter M., Steininger C., Vossen M.G., Winkler S., Thalhammer F. (2018). Dalbavancin as Primary and Sequential Treatment for Gram-Positive Infective Endocarditis: 2-Year Experience at the General Hospital of Vienna. Clin. Infect. Dis..

[22] Escolà-Vergé L., Fernández-Hidalgo N., Rodríguez-Pardo D., Pigrau C., González-López J.J., Bartolomé R., Almirante B. (2019). Teicoplanin for treating enterococcal infective endocarditis: A retrospective observational study from a referral centre in Spain. Int. J. Antimicrob. Agents.

[23] De Nadaï T., François M., Sommet A., Dubois D., Metsu D., Grare M., Marchou B., Delobel P., Martin-Blondel G. (2019). Efficacy of teicoplanin monotherapy following initial standard therapy in Enterococcus faecalis infective endocarditis: A retrospective cohort study. Infectction.

[24] Suzuki H., Carlson J.R., Matsumoto E. (2019). Treatment of Enterococcus faecalis infective endocarditis with penicillin G plus ceftriaxone. Infect. Dis. (Auckl.).

[25] Tritle B.J., Timbrook T.T., A Fisher M., Spivak E.S. (2019). Penicillin as a Potential Agent for Dual Beta-lactam Therapy for Enterococcal Endocarditis. Clin. Infect. Dis..

[26] Gavaldà J., Len O., Miró J.M., Muñoz P., Montejo M., Alarcón A., De La Torre-Cisneros J., Peña C., Martínez-Lacasa X., Sarriá C. (2007). Brief Communication: Treatment of Enterococcus faecalis Endocarditis with Ampicillin plus Ceftriaxone. Ann. Intern. Med..

[27] Pericas J.M., Cervera C., Del Rio A., Moreno A., De La Maria C.G., Almela M., Falces C., Ninot S., Castaneda X., Armero Y. (2014). Changes in the treatment of Enterococcus faecalis infective endocarditis in Spain in the last 15 years: From ampicillin plus gentamicin to ampicillin plus ceftriaxone. Clin. Microbiol. Infect..

[28] Carugati M., Bayer A.S., Miró J.M., Park L.P., Guimarães A.C., Skoutelis A., Fortes C.Q., Durante-Mangoni E., Hannan M.M., Nacinovich F. (2013). High-Dose Daptomycin Therapy for Left-Sided Infective Endocarditis: A Prospective Study from the International Collaboration on Endocarditis. Antimicrob. Agents Chemother..

[29] Pericas J.M., Cervera C., Moreno A., García-De-La-Mària C., Almela M., Falces C., Quintana E., Vidal B., Llopis J., Fuster D. (2018). Outcome of Enterococcus faecalis infective endocarditis according to the length of antibiotic therapy: Preliminary data from a cohort of 78 patients. PLoS ONE.

[30] El Rafei A., DeSimone D.C., Narichania A.D., Sohail M.R., Vikram H.R., Li Z., Steckelberg J.M., Wilson W.R., Baddour L.M. (2018). Comparison of Dual β-Lactam therapy to penicillin-aminoglycoside combination in treatment of Enterococcus faecalis infective endocarditis. J. Infect..

[31] Fernández-Hidalgo N., Almirante B., Gavaldà J., Gurgui M., Peña C., De Alarcón A., Ruiz J., Vilacosta I., Montejo M., Vallejo N. (2013). Ampicillin Plus Ceftriaxone Is as Effective as Ampicillin Plus Gentamicin for TreatingEnterococcus faecalisInfective Endocarditis. Clin. Infect. Dis..

[32] Bassetti M., Russo A., Givone F., Ingani M., Graziano E., Bassetti M. (2019). Should High-dose Daptomycin be an Alternative Treatment Regimen for Enterococcal Endocarditis?. Infect. Dis. Ther..

[33] Dahl A., Rasmussen R.V., Bundgaard H., Hassager C., Bruun L.E., Lauridsen T.K., Moser C., Sogaard P., Arpi M., Bruun N.E. (2013). Enterococcus faecalis Infective Endocarditis. Circulation.

[34] Hidalgo-Tenorio C., Vinuesa D., Plata A., Dávila P.M., Iftime S., Sequera S., Loeches B., López-Azkarreta Í., Fariñas M.C., Fernández-Roldan C. (2019). DALBACEN cohort: Dalbavancin as consolidation therapy in patients with endocarditis and/or bloodstream infection produced by gram-positive cocci. Ann. Clin. Microbiol. Antimicrob..

[35] Presterl E., Graninger W., Georgopoulos A. (1993). The efficacy of teicoplanin in the treatment of endocarditis caused by Gram-positive bacteria. J. Antimicrob. Chemother..

[36] Lebeaux D., Fernández-Hidalgo N., Pilmis B., Tattevin P., Mainardi J.-L. (2019). Aminoglycosides for infective endocarditis: Time to say goodbye?. Clin. Microbiol. Infect..

[37] Olaison L. (2002). Enterococcal Endocarditis in Sweden, 1995–1999: Can Shorter Therapy with Aminoglycosides Be Used?. Clin. Infect. Dis..

[38] Maher M., Jensen K.J., Lee D., Nix D.E. (2016). Stability of Ampicillin in Normal Saline and Buffered Normal Saline. Int. J. Pharm. Compd..

[39] Kang M.A., Kang J.-S. (2012). Stability Test of Ampicillin Sodium Solutions in the Accufuser® Elastomeric Infusion Device Using HPLC: UV Method. Pharmacol. Pharm..

[40] Herrera-Hidalgo L., López-Cortes L.E., Luque-Márquez R., Gálvez-Acebal J., De Alarcón A., Gutiérrez-Valencia A., Gil-Navarro M.V., López-Cortes L.F. (2020). Ampicillin and Ceftriaxone Solution Stability at Different Temperatures in Outpatient Parenteral Antimicrobial Therapy. Antimicrob. Agents Chemother..

[41] Gavaldà J., Torres C., Tenorio C., López P., Zaragoza M., Capdevila J.A., Almirante B., Ruiz F., Borrell N., Gomis X. (1999). Efficacy of Ampicillin plus Ceftriaxone in Treatment of Experimental Endocarditis Due to Enterococcus faecalis Strains Highly Resistant to Aminoglycosides. Antimicrob. Agents Chemother..

[42] Liao C.-H., Huang Y.-T., Tsai H.-Y., Hsueh P.-R. (2014). In vitro synergy of ampicillin with gentamicin, ceftriaxone and ciprofloxacin against Enterococcus faecalis. Int. J. Antimicrob. Agents.

[43] Spellberg B., Chambers H.F., Musher D.M., Walsh T.L., Bayer A.S. (2020). Evaluation of a Paradigm Shift From Intravenous Antibiotics to Oral Step-Down Therapy for the Treatment of Infective Endocarditis. JAMA Intern. Med..

[44] Kobayashi T., Ando T., Streit J., Sekar P. (2019). Current Evidence on Oral Antibiotics for Infective Endocarditis: A Narrative Review. Cardiol. Ther..

[45] Colli A., Campodonico R., Gherli T. (2007). Early Switch From Vancomycin to Oral Linezolid for Treatment of Gram-Positive Heart Valve Endocarditis. Ann. Thorac. Surg..

[46] Muñoz P., Rodríguez-Creixems M., Moreno M., Marin M., González-Ramallo V., Bouza E., Garcia-Pavia P., GAME Study Group (2007). Linezolid therapy for infective endocarditis. Clin. Microbiol. Infect..

[47] Fernández-Hidalgo N., Escolà-Vergé L., Pericas J.M. (2020). Enterococcus faecalis endocarditis: What’s next?. Future Microbiol..

[48] Moher D., Liberati A., Tetzlaff J., Altman U.G. (2009). Preferred reporting items for systematic reviews and meta-analyses: The PRISMA statement. BMJ.

[49] Schünemann H.J., Tugwell P., Reeves B.C., Akl E.A., Santesso N., Spencer F.A., Shea B., Wells G., Helfand M. (2013). Non-randomized studies as a source of complementary, sequential or replacement evidence for randomized controlled trials in systematic reviews on the effects of interventions. Res. Synth. Methods.

[50] Higgins J.P.T., Altman U.G., Gøtzsche P.C., Jüni P., Moher D., Oxman A.D., Savović J., Schulz K.F., Weeks L., Sterne J.A.C. (2011). The Cochrane Collaboration’s tool for assessing risk of bias in randomised trials. BMJ.

[51] Sterne J.A.C., Hernán M.A., Reeves B.C., Savović J., Berkman N.D., Viswanathan M., Henry D., Altman D.G., Ansari M.T., Boutron I. (2016). ROBINS-I: A tool for assessing risk of bias in non-randomised studies of interventions. BMJ.

[52] NIH Study Quality Assessment Tools n.d. https://www.nhlbi.nih.gov/health-topics/study-quality-assessment-tools.

[53] Ryan R. Group Cochrane Consumers and Communication Review. Cochrane Consumers and Communication Review Group: Data synthesis and analysis n.d. https://cccrg.cochrane.org/.

